# Potential causal and temporal relationship between plasma triglyceride levels and circulating leukocyte

**DOI:** 10.1016/j.jlr.2024.100662

**Published:** 2024-10-05

**Authors:** Jing Xian Fang, Hui Min Zou, Jian Meng, Yu Han, Xue Hu, Qing Gu, Sui Jun Wang, Xing Zhen Liu

**Affiliations:** 1 Department of Endocrinology, Shidong Hospital, University of Shanghai for Science and Technology, Shanghai, China; 2 Hangzhou Aeronautical Sanatorium for Special Service of China Air Force, Hangzhou, China

**Keywords:** triglyceride, dyslipidemia, Mendelian randomization, leukocyte, cross-lagged panel model

## Abstract

Circulating triglyceride (TG) and leukocytes, the main components of the vascular system, may impact each other and co-fuel atherosclerosis. While the causal relationship between plasma TG levels and leukocyte counts remains unclear. Bidirectional Mendelian randomization (MR) analysis was conducted to investigate the potential causal relationship between TG levels and the counts of leukocytes and their subtypes. A cross-lagged panel model (CLPM) using longitudinal healthy screening data (13,389 adults with a follow-up of 4 years) was fitted to examine the temporal relationship between them. Genetically predicted plasma TG levels were positively associated with total leukocyte counts (TLC) [β(se) = 0.195(0.01)], lymphocyte counts (LC) [β(se) = 0.196(0.019)], and neutrophil counts (NC) [β(se) = 0.086(0.01)], which remained significant after adjusting for several confounders. Inversely, the genetically predicted TLC [β(se) = 0.033(0.008)], LC [β(se) = 0.053(0.008)], and NC [β(se) = 0.034(0.008)] were positively associated with plasma TG levels. However, when all three of them were put into the MR model adjusted for each other, only LC was significantly associated with TG levels. There was no association between genetically predicted TG levels and monocyte counts (MC), basophil counts, and eosinophil counts. The results of CLPM showed that the temporal effect of elevated TLC, MC, LC, and NC on plasma TG levels was stronger than the inverse effect. Our findings suggest causal associations of plasma TG levels with TLC, LC, and NC. In turn, LC was positively associated with plasma TG levels. Additionally, elevated circulating LC may precede high plasma TG levels.

Dyslipidemia is a well-established risk factor for the initiation and development of atherosclerotic cardiovascular disease (ASCVD) (1). In addition to the direct involvement of excess cholesterol accumulation in the subendothelium of blood vessels in the formation of atheromatous plaques, local or systemic inflammation induced by other factors is also thought to be a major pathologic mechanism of ASCVD (2, 3). This has spawned more research on the involvement of immune cells in the initiation, development, and rupture of atheromatous plaques by mediating inflammation (4, 5).

The critical role of dyslipidemia-driven inflammation in atherosclerosis was noted several decades ago, which has also brought attention to the association between lipids, immune cells, cardiovascular risk factors, and ASCVD (6, 7, 8). TG-leukocyte interactions in the subendothelial space of the vasculature, adipose tissue, and bone marrow are well established (9, 10). In the bloodstream, a close relationship between plasma TG levels and circulating leukocytes (economic and simple clinical indicators) has also been established by several observational studies (11, 12). Nevertheless, whether there is a causal association between plasma TG levels and circulating leukocyte counts and the direction of the association is still unclear.

In this study, bidirectional Mendelian randomization (MR) using publicly available genome-wide association study (GWAS) data was adopted to assess the potential causal relationship between circulating triglyceride (TG) levels and the counts of leukocytes and their subtypes. Furthermore, a cross-lagged panel model (CLPM) using longitudinal healthy screening data of Chinese adults was fitted to investigate the temporal relationship between them. We hope this study will help us better understand the interaction between dyslipidemia and immune cells, and provide more insights into the pathological mechanism of ASCVD.

## Materials and Methods

### Data sources

The summary-level GWAS data were retrieved from the publicly available datasets of the MRC Integrative Epidemiology Unit (IEU) OpenGWAS data infrastructure at the University of Bristol (https://gwas.mrcieu.ac.uk). The GWAS data for circulating concentration of TG, low-density lipoprotein cholesterol (LDLc), and apolipoprotein B (Apo B) were obtained from the UK Biobank database (n = 441,016) (13). The GWAS data for total leukocyte counts (TLC), monocyte counts (MC), lymphocyte counts (LC), neutrophill counts (NC), basophil counts (BC), and eosinophil counts (EC) was obtained from Chen MH *et al.*’s bloob cell consortium (n = 562,243, indicated as C1 in the supplementary Tables) (14). Meanwhile, GWAS data for blood cell traits from Mbatchou J *et al.*’s blood cell consortium (n = 396,621, indicated as C2 in the supplementary Tables) were also used in the MR analysis for sensitivity analysis (15). The GIANT consortium’s summary GWAS data (n = 681,275) for body mass index (BMI) were used (16). The GWAS data for the concentration of very-low-density lipoproteins (VLDL) particles was obtained from Richardson TG *et al.*’s GWAS (115,082 UK Biobank participants) (17). Details of summary GWAS data sources used for this MR analysis are shown in Supplemental Table S1A. Due to the summary-level GWAS data we used are publicly available, no additional written informed consent was obtained from all participants.

Longitudinal data were obtained from the general population of those attending annual health screenings rather than outpatient visits or hospitalizations. (January 2013-March 2024, general Han population in eastern China). A total of 13,389 adults with a follow-up of 4 years were used to explore the temporal relationship between plasma TG levels and circulating leukocytes. Those individuals who were in the acute phase of the disease (upper respiratory tract infections, pneumonia, viral hepatitis B, acute attacks of gout or allergic diseases, etc.) and/or taking medications that affect the levels of lipids and blood cell parameters (antibiotics, glucocorticosteroids, or lipid regulating drugs, etc.) were excluded. The basic characteristics at baseline and follow-up are shown in Supplemental Table S1B. The use of these data and study protocols was approved by the ethics committee of Shanghai Shidong Hospital.

### Genetic variants selection

The single nucleotide polymorphisms (SNPs) that are associated with traits at the level of genome-wide significance (*P* < 5 × 10^−8^) were retrieved from the corresponding GWAS summary-level dataset. The linkage disequilibrium (LD) statistics (*r*^2^) were evaluated to ensure the independence among selected SNPs. The thresholds of LD-clumping were set based on the LD information provided in the original literature of GWAS.

The ‘TwoSampleMR’ (v0.5.6) R package was used to select and extract the details of each SNP, which are shown in Supplemental Table S2. The SNPs that are both associated with TG and leukocytes will be excluded from subsequent MR analysis (SNPs marked in yellow in Supplemental Table S2). Steiger filtering was also conducted to test potential reverse causation for the selected SNPs, and the results of Steiger filtering are shown in Supplemental Table S3. The SNPs strongly associated with the outcomes were similarly eliminated from the MR analysis. The number of selected SNPs used in each MR analysis was presented in the corresponding MR results.

### Statistical analysis

Bidirectional MR analysis to examine the causal relationship between circulating TG and leukocytes. Prior to this, the Steiger test was performed to initially assess the direction of causality between them. As shown in Supplemental Table S4, there appears to be a bidirectional causal relationship between circulating TG and leukocytes. In subsequent specific MR analyses, the inverse variance weighted (IVW) method (multiplicative random-effects model) was used as the primary MR approach (18). Results were reported as beta-coefficients (β value) [SD unit of outcomes per SD unit change in exposures]. Three additional MR approaches of MR-Egger, Weighted Median, and MR-PRESSO were used as sensitivity analyses. The MR-Egger method can be used to detect horizontal pleiotropy (*P* value for its intercept) and to preclude pleiotropy after adjusting for pleiotropic effects (19). The weighted median model can provide consistent causal estimates (20). The MR-PRESSO estimator approach detects outlying SNPs and makes estimates after the removal of potential outliers (21).

In addition to the univariate MR (UVMR), multivariable MR (MVMR) analysis was also performed to adjust for potential confounders (BMI, LDLc, VLDL, and Apo B) (22). An association between exposure and outcome was considered significant if the result of the IVW method was significant, and the directions of effect obtained by the other MR methods were consistent with those of the IVW method.

Generally, a *P*-value of less than 0.05 was considered statistically significant. To be less subject to the effects of multiple testing, a Bonferroni-corrected significance threshold (*P* < 0.05/n, n = the number of traits at each stage of the MR analysis) was used as a correction for multiple testing. MR analysis was conducted using the R packages of ‘MendelianRandomization’ and ‘Mendelian Randomization Pleiotropy RESidual Sum and Outlier’ (‘MRPRESSO’). Reporting of MR results followed the STROBE-MR Statement (23).

The cross-lagged panel analysis was performed to explore the temporal relationship between TG and the counts of leukocytes and their subtypes. When building the CLPM, the models were adjusted for baseline age, sex, BMI, and LDLc. The validity of model fitting was indicated by root mean square residual (RMR) and comparative fit index (CFI). RMR<0.05 and CFI>0.90 indicated a relatively good fit to the observed data (24). All statistical calculations and the production of figures were performed using R (version 4.2.1, The R Foundation for Statistical Computing).

## Results

### Associations between plasma TG levels and circulating leukocyte counts

**Fig. 1:**
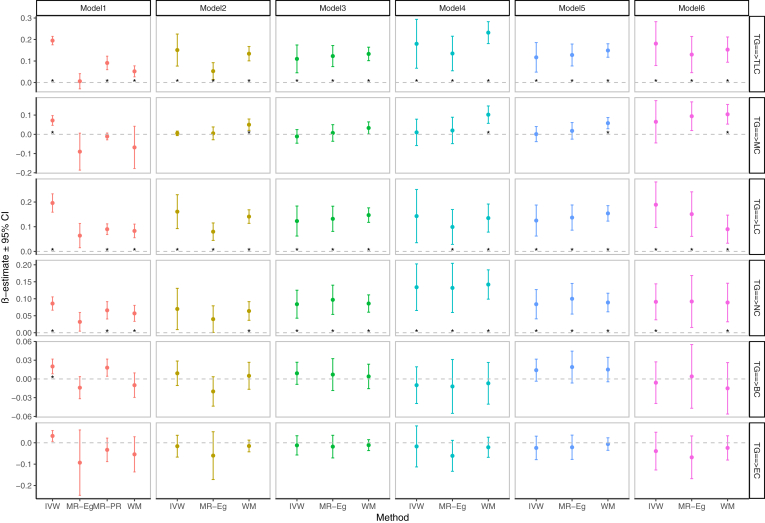
Association of genetically predicted concentration of TG with circulating leukocyte counts. MR results are reported as β value with 95% CI (SD unit of outcomes per SD unit change in exposures); units of traits and abbreviations are presented in Supplemental Table S1A; Details of MR results can be found in Supplemental Tables S1–S5 to Supplemental Tables S5–S6; Model 1 was not adjusted, Models 2-6 adjusted for BMI, LDLc, VLDL, Apo B, and all of them, respectively; the asterisks indicate the Bonferroni-corrected significance threshold [0.0083(0.05/6)].

The genetically predicted increase in plasma TG levels was associated with higher TLC [IVW: β(se) = 0.195(0.01), *P* < 0.001], LC [IVW: β(se) = 0.196(0.019), *P* < 0.001], and NC [IVW: β(se) = 0.086(0.01), *P* < 0.001]. The significant IVW causal estimates detected above were supported by the other three MR methods (Fig. 1 and Supplemental Tables S1–S5). In MVMR analysis, the associations of genetically determined plasma TG concentration with higher TLC, LC, and NC were still significant when BMI, LDLc, VLDL, Apo B, and all of them were adjusted. (Fig. 1 and Supplemental Tables S2–S5 to Supplemental Tables S5 and S6).

**Fig. 2:**
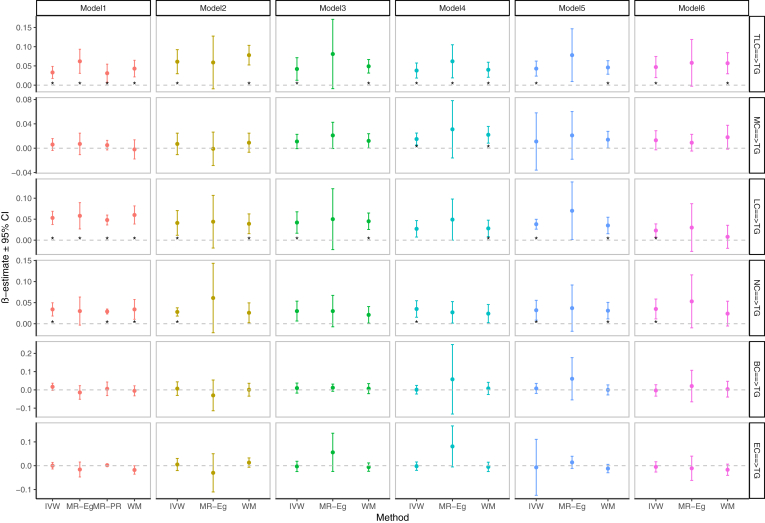
Association of genetically predicted circulating leukocyte counts with plasma TG levels. MR results are reported as β value with 95% CI (SD unit of outcomes per SD unit change in exposures); units of traits and abbreviations are presented in Supplemental Table S1A; Details of MR results can be found in Supplemental Tables S1–S6 to Supplemental Table S6; Model 1 was not adjusted, Models 2-6 adjusted for BMI, LDLc, VLDL, Apo B, and all of them, respectively; the asterisks indicate the Bonferroni-corrected significance threshold [0.0083(0.05/6)].

In the reverse MR analysis, the genetically predicted higher TLC [IVW: β(se) = 0.033(0.008), *P* < 0.001], LC [IVW: β(se) = 0.053(0.008), *P* < 0.001], and NC [IVW: β(se) = 0.034(0.008), *P* < 0.001] were associated with elevated plasma TG levels. The significant IVW causal estimates detected above were supported by the other three MR methods (Fig. 2 and Supplemental Tables S5–S7). In MVMR analysis, the association of genetically determined TLC, LC, and NC with plasma TG concentration was still significant when adjusting for BMI, LDLc, VLDL, and Apo B, either individually or simultaneously (Fig. 2 and Supplemental Tables S5–S8 to Supplemental Tables S5–S12). Current MR analysis did not show a significant association between TG levels and MC, BC, and EC.

**Fig. 3:**
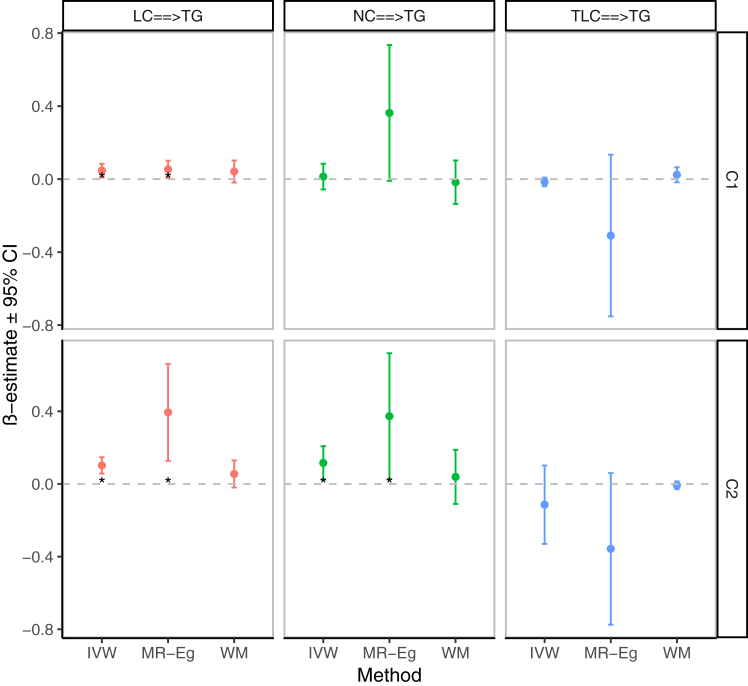
Association of genetically predicted TLC, LC, and NC with plasma TG levels after adjusting them to each other. MR results are reported as β value with 95% CI (SD unit of outcomes per SD unit change in exposures); units of traits and abbreviations are presented in Supplemental Table S1A; Details of MR results can be found in Supplemental Tables S5–13 and Supplemental S6–S13; C1: the summary-level GWAS data on blood cell traits from Chen MH *et al.*’s blood cell consortium (PMID: 32888493); C2: the summary-level GWAS data on blood cell traits from Mbatchou J *et al.*’s blood cell consortium (PMID: 34017140); the asterisks indicate the Bonferroni-corrected significance threshold [0.0167(0.05/3)].

When TLC, LC, and NC were all put into the MVMR model adjusted for each other, only LC was significantly associated with TG levels (Fig. 3 and Supplemental Tables S5–S13). As shown in Supplemental Table S6, the current results were in general accordance with the results of the MR analysis utilizing another summary-level GWAS data of blood cell traits (PMID: 34017140). Notably, unlike the previous blood cell consortium, both LC and NC were significantly associated with TG levels after TLC, LC, and NC were put into the MVMR model simultaneously. (Fig. 3 and Supplemental Tables S6–S13).

### Temporal relationships between TG levels and leukocyte counts

**Fig. 4:**
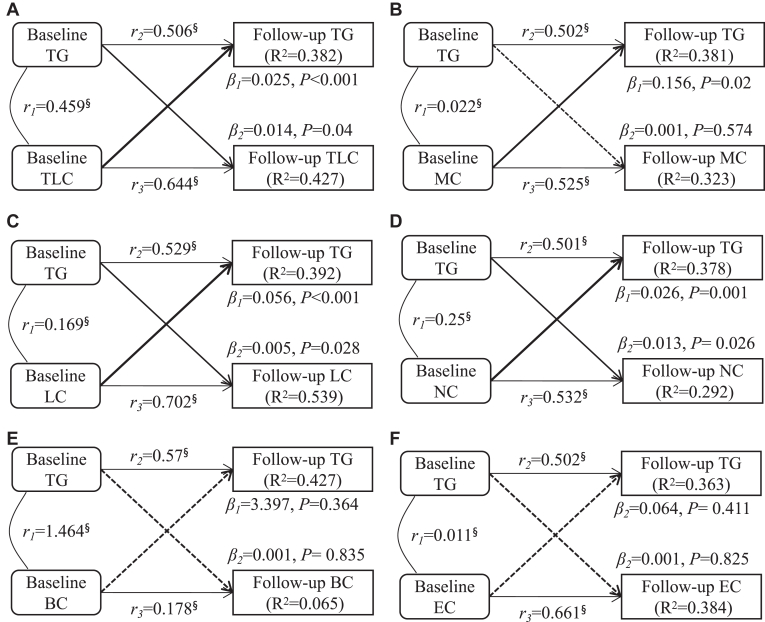
Temporal relationships between plasma TG levels and leukocytes counts. Results were adjusted for baseline age, sex, BMI, and LDLc; β_1_, cross-lagged path coefficients from baseline leukocyte counts to follow-up plasma TG levels; β_2_, cross-lagged path coefficients from baseline plasma TG levels to follow-up leukocyte counts; r_1_, synchronous correlation; r_2_ and r_3_, tracking correlations; *R*^2^, variance explained; abbreviations can be found in Supplemental Table S1A; the dashed line indicated that the β-value was not statistically significant; a solid line, on the other hand, indicated statistical significance, with a thicker solid line indicating that the β-value in that direction is greater than the other direction's.

The results of cross-lagged panel analysis of TG and circulating leukocyte counts, adjusting for baseline age, sex, BMI, and LDLc, are shown in Fig. 4. The path coefficients (β_1_) of baseline TLC (β_1_ = 0.025, *P* < 0.001, Fig. 4A), MC (β_1_ = 0.25, *P* < 0.001, Fig. 4B), LC (β_1_ = 0.056, *P* < 0.001, Fig. 4C), and NC (β_1_ = 0.026, *P* = 0.001, Fig. 4D) to the follow-up TG were significantly greater than the path coefficient (β_2_) from baseline TG to follow-up TLC (β_2_ = 0.014, *P* = 0.04), MC (β_2_ = 0.002, *P* = 0.013), LC (β_2_ = 0.005, *P* = 0.028), and NC (β_2_ = 0.013, *P* = 0.026). In the TG ↔ BC and TG ↔ EC models analysis, the path coefficients (β_1_ and β_2_) were not significant (Fig. 4E and F). Model fitting parameters were RMR≤0.07 and CFI≥0.86 in all models.

## Discussion

In the MR framework, we investigated the potential causal associations between plasma TG levels and circulating leukocyte counts. We found a causal association of TG levels with the counts of total leukocytes, lymphocytes, and neutrophils. The reverse MR analysis showed that lymphocyte counts were positively associated with plasma TG levels. Furthermore, a CLPM using a longitudinal cohort was fitted to explore the temporal relationship between plasma TG levels and circulating leukocyte counts. The results showed that the temporal effect of elevated counts of total leukocytes, lymphocytes, and neutrophils on plasma TG levels was stronger than the inverse effect, which suggests that elevated circulating lymphocyte counts may precede the elevation of plasma TG levels, although there may be a bidirectional causal relationship between them.

The link between plasma TG levels and leukocyte counts has been established by several observational and experimental studies (9, 10). However, whether there is a causal association between TG levels and leukocyte counts and the direction of causality has not been well explored. This is largely attributable to the fact that observational studies are inevitably subject to confounding factors and reverse causation when explaining causal relationships. In contrast, MR design can be less vulnerable to confounding and reverse causation bias, and thus reliably show potential causality (25). Thus the current findings based on MR analysis provide strong evidence for the bidirectional interaction between dyslipidemia and immune cells.

Consistent with what has been reported in the vast majority of observational studies, we observed a positive association between plasma TG levels and total leukocyte counts. When exploring the relationship between TG levels and different leukocyte subtypes, however, different conclusions were observed. A study by Tucker *et al.* using data from the UK Biobank cohort identified a positive association between TG levels and the circulating counts of all five leukocyte subtypes (26). Another study using data from the United States National Health and Nutritional Examination Survey found that the association between TG levels and different leukocyte subtypes was different across sex (27).

In the present study, we similarly observed inconsistent associations between TG levels and all five leukocyte subtypes. We found that TG levels were only causally associated with the counts of lymphocytes and neutrophils. Neutrophils and lymphocytes play important roles in innate and adaptive cellular immune responses (28). Their homeostasis is a balance between production, proliferation, and death, which is influenced by co-stimulatory molecules, cytokines, and metabolic situations (29). Although the exact mechanism remains unclear, elevated circulating TG may affect neutrophil and lymphocyte homeostasis by increasing free fatty acids (FFA) and lipid accumulation of bone marrow (30, 31).

In addition to immune defense, leukocytes have been implicated in the pathogenesis of autoimmune diseases, dyslipidemia, and ASCVD as key pro-inflammatory mediators (32, 33). To determine whether circulating leukocyte counts, in turn, have an effect on TG levels, we performed reverse MR analysis. The results showed that lymphocyte counts were positively associated with TG levels after adjusting for some confounding factors (including other leukocyte subtypes), which was consistent with several clinical observations (34). Elevated lymphocyte counts may affect plasma TG levels through the following mechanisms. First, the suppression of lipoprotein lipase (LPL) by proinflammatory factors released by increased circulating lymphocytes and neutrophils may result in higher levels of plasma TG (35, 36). Second, infiltration of excess circulating lymphocytes and neutrophils into adipose tissue will promote lipolysis and increase circulating FFA levels, which thus increases hepatic TG synthesis and release into the bloodstream (37, 38).

Notably, the neutrophil effect on TG levels was maintained when adjusting for common confounding factors. However, when the counts of total leukocytes, lymphocytes, and neutrophils were entered into the model at the same time (adjusted for each other), the effect of neutrophils on TG levels became erratic. It is difficult to explain this result, but it might be related to the fact that neutrophils share some regulatory genes with lymphocytes. In addition, synergistic interactions between lymphocytes and neutrophils can activate the formation of neutrophil extracellular traps (NETs) (39). Both the 3-dimensional meshwork of NETs and the enzymes within it may have a pronounced effect on circulating lipids, although this is not yet well characterized (40).

Having clarified that there may be a ‘vicious circle’ between high TG levels and elevated lymphocyte counts, we are very curious as to who is the forerunner of this ‘vicious cycle’. We therefore used longitudinal observational data and CLPM to try to answer this question. The results suggest that high circulating lymphocyte counts may precede the elevation of plasma TG levels. We speculate that this may be related to the fact that lymphocytes have a more rapid mobilization rate, which is in response to injury and pathogen invasion (41). In the case of obesity or vascular endothelial injury, elevated local or precirculatory proinflammatory factors can rapidly mobilize immune cells into circulation (42).

Unlike leukocytes, TG serves primarily to store energy and exists in many forms. In adipose tissue or the liver, TG are found in the form of lipid droplets (43). In circulation, TG needs the help of lipoproteins to exist in the form of chylomicrons or VLDL, which are secreted by the intestine and liver, respectively (44). Thus, plasma TG levels may be influenced by additional factors such as LPL activity, FFA turnover, diet, and the liver's ability to synthesize VLDL. Therefore, characteristic differences between leukocytes and TG may explain to some extent the elevation of circulating counts of lymphocytes and neutrophils precede high plasma TG levels.

Eosinophils and basophils primarily mediate allergic inflammation (45). So it is not surprising that no associations between TG levels and counts of eosinophil and basophil were observed in this study. Unexpectedly, our MR analysis did not reveal a potential causal association between TG levels and monocyte counts. After all, monocytes and the macrophages they differentiate into are crucial cells in the formation of atheromatous plaques (46).

The absence of an association between monocyte counts and TG levels has also been reported by several other observational studies (10). The relationships between monocyte counts and TG levels may partly be explained by the shorter residence time of monocytes in the peripheral blood in a number of pathological conditions, such as systemic inflammation, obesity, or vascular endothelial injury. Saja *et al.* (47) found that hypertriglyceridemia promotes extravasation of monocytes and tissue macrophages accumulation.

The present study is the first, to our knowledge, to combine GWAS and longitudinal observational data to explore the relationship between plasma TG levels and circulating leukocytes. These findings can help us better understand the interactions between leukocytes and lipids, two of the most important components of the vasculature. While the generalizability of the current findings may be subject to certain limitations. First, the summary-level GWAS data used made it impossible to assess whether covariates (dietary habits and basic diseases) had any effect on the results. Second, although multiple sensitivity analyses support the primary findings, pleiotropy remains an unavoidable problem in the interpretation of MR results. Third, the GWAS data we used all originated from Europe, but the longitudinal data were obtained from the Han Chinese population. These racial-specific data may hamper the generalization of the findings to populations of other ancestries. Fourth, the use of two-sample MR may lead to some bias due to the unavoidable sample overlap attributed to the fact that most of the GWAS data come from UKB.

In conclusion, we clarified that there is a causal association of plasma TG levels with the counts of total leukocyte, lymphocyte, and neutrophil. In turn, lymphocyte counts were found to be causally associated with plasma TG levels. Moreover, elevated counts of lymphocytes are more likely to precede high TG levels. These findings provide novel evidence for the interaction between circulating immune cells and lipids. In addition, the lymphocyte count, an economical and simple clinical test, may be used as a monitor for dyslipidemia.

## Data availability

The GWAS data used during the current study are publicly available. Longitudinal data and statistical analysis codes are available from the corresponding author on reasonable request.

## Supplemental data

This article contains supplemental data.

## Ethics approval and consent to participate

Because the GWAS data used in current MR study are publicly available, no additional written informed consent was obtained from all participants. The use of longitudinal data and study protocols were approved by the ethics committee of Shanghai Shidong Hospital. Because the longitudinal data was collected retrospectively, the need to obtain informed consent from eligible patients was waived by the ethics committee.

## Consent for publication

All authors agree to publish this work.

## Conflict of interests

The authors declare that they have no conflicts of interest with the contents of this article.

## References

[1] Hussain I., Patni N., Garg A. (2019). Lipodystrophies, dyslipidaemias and atherosclerotic cardiovascular disease. Pathology.

[2] Henein M.Y., Vancheri S., Longo G., Vancheri F. (2022). The role of inflammation in cardiovascular disease. Int. J. Mol. Sci..

[3] Bäck M., Yurdagul A., Tabas I., Öörni K., Kovanen P.T. (2019). Inflammation and its resolution in atherosclerosis: mediators and therapeutic opportunities. Nat. Rev. Cardiol..

[4] Poller W.C., Nahrendorf M., Swirski F.K. (2020). Hematopoiesis and cardiovascular disease. Circ. Res..

[5] Jaiswal S., Ebert B.L. (2019). Clonal hematopoiesis in human aging and disease. Science.

[6] Morgan P.K., Fang L., Lancaster G.I., Murphy A.J. (2020). Hematopoiesis is regulated by cholesterol efflux pathways and lipid rafts: connections with cardiovascular diseases. J. Lipid Res..

[7] Liu L.Y., Gu Q., Hu X., Fan J., Liu X.Z. (2022). Potential mediators of causal associations of circulating triglycerides with blood pressure: evidence from genetic and observational data. Hypertension.

[8] Siedlinski M., Jozefczuk E., Xu X., Teumer A., Evangelou E., Schnabel R.B. (2020). White blood cells and blood pressure: a mendelian randomization study. Circulation.

[9] Madsen S., Ramosaj M., Knobloch M. (2021). Lipid metabolism in focus: how the build-up and breakdown of lipids affects stem cells. Development.

[10] Gisterå A., Hansson G.K. (2017). The immunology of atherosclerosis. Nat. Rev. Nephrol..

[11] Sawant S., Tucker B., Senanayake P., Waters D.D., Patel S., Rye K.A. (2021). The association between lipid levels and leukocyte count: a cross-sectional and longitudinal analysis of three large cohorts. Am. Heart J. Plus.

[12] Lai Y.C., Woollard K.J., McClelland R.L., Allison M.A., Rye K.A., Ong K.L. (2019). The association of plasma lipids with white blood cell counts: results from the Multi-Ethnic Study of Atherosclerosis. J. Clin. Lipidol..

[13] Richardson T.G., Sanderson E., Palmer T.M., Ala-Korpela M., Ference B.A., Davey Smith G. (2020). Evaluating the relationship between circulating lipoprotein lipids and apolipoproteins with risk of coronary heart disease: a multivariable Mendelian randomisation analysis. PloS Med..

[14] Chen M.H., Raffield L.M., Mousas A., Sakaue S., Huffman J.E., Moscati A. (2020). Trans-ethnic and ancestry-specific blood-cell genetics in 746,667 individuals from 5 global populations. Cell.

[15] Mbatchou J., Barnard L., Backman J., Marcketta A., Kosmicki J.A., Ziyatdinov A. (2021). Computationally efficient whole-genome regression for quantitative and binary traits. Nat. Genet..

[16] Yengo L., Sidorenko J., Kemper K.E., Zheng Z., Wood A.R., Weedon M.N., GIANT Consortium (2018). Meta-analysis of genome-wide association studies for height and body mass index in ∼700000 individuals of European ancestry. Hum. Mol. Genet..

[17] Richardson T.G., Leyden G.M., Wang Q., Bell J.A., Elsworth B., Davey Smith G. (2022). Characterising metabolomic signatures of lipid-modifying therapies through drug target mendelian randomisation. PloSBiol..

[18] Burgess S., Butterworth A., Thompson S.G. (2013). Mendelian randomization analysis with multiple genetic variants using summarized data. Genet. Epidemiol..

[19] Bowden J., Davey Smith G., Burgess S. (2015). Mendelian randomization with invalid instruments: effect estimation and bias detection through Egger regression. Int. J. Epidemiol..

[20] Bowden J., Davey Smith G., Haycock P.C., Burgess S. (2016). Consistent estimation in mendelian randomization with some invalid instruments using a weighted median estimator. Genet. Epidemiol..

[21] Verbanck M., Chen C.Y., Neale B., Do R. (2018). Detection of widespread horizontal pleiotropy in causal relationships inferred from Mendelian randomization between complex traits and diseases. Nat. Genet..

[22] Rasooly D., Peloso G.M. (2021). Two-sample multivariable mendelian randomization analysis using R. Curr. Protoc..

[23] Skrivankova V.W., Richmond R.C., Woolf B.A.R., Yarmolinsky J., Davies N.M., Swanson S.A. (2021). Strengthening the reporting of observational studies in Epidemiology using mendelian randomization: the STROBE-MR statement. JAMA.

[24] Falkenström F., Solomonov N., Rubel J. (2020). Using time-lagged panel data analysis to study mechanisms of change in psychotherapy research: methodological recommendations. Couns. Psychother. Res..

[25] Larsson S.C., Butterworth A.S., Burgess S. (2023). Mendelian randomization for cardiovascular diseases: principles and applications. Eur. Heart J..

[26] Tucker B., Sawant S., McDonald H., Rye K.A., Patel S., Ong K.L. (2021). The association of serum lipid and lipoprotein levels with total and differential leukocyte counts: results of a cross-sectional and longitudinal analysis of the UK Biobank. Atherosclerosis.

[27] Andersen C.J., Vance T.M. (2019). Gender dictates the relationship between serum lipids and leukocyte counts in the national health and nutrition examination Survey 1999-2004. J. Clin. Med..

[28] Costa S., Bevilacqua D., Cassatella M.A., Scapini P. (2019). Recent advances on the crosstalk between neutrophils and B or T lymphocytes. Immunology.

[29] Krammer P.H., Arnold R., Lavrik I.N. (2007). Life and death in peripheral T cells. Nat. Rev. Immunol..

[30] Ortega-Gómez A., Varela L.M., López S., Montserrat de la Paz S., Sánchez R., Muriana F.J.G. (2017). Postprandial triglyceride-rich lipoproteins promote lipid accumulation and apolipoprotein B-48 receptor transcriptional activity in human circulating and murine bone marrow neutrophils in a fatty acid-dependent manner. Mol. Nutr. Food Res..

[31] Ortega-Gomez A., Lopez S., Varela L.M., Jaramillo S., Muriana F.J.G., Abia R. (2022). New evidence for dietary fatty acids in the neutrophil traffic between the bone marrow and the peripheral blood. Food Chem. (Oxf).

[32] Luo J., Thomassen J.Q., Nordestgaard B.G., Tybjærg-Hansen A., Frikke-Schmidt R. (2023). Neutrophil counts and cardiovascular disease. Eur. Heart J..

[33] Liu Y., Kong X., Wang W., Fan F., Zhang Y., Zhao M. (2017). Association of peripheral differential leukocyte counts with dyslipidemia risk in Chinese patients with hypertension: insight from the China Stroke Primary Prevention Trial. J. Lipid Res..

[34] Phillips A.C., Carroll D., Gale C.R., Drayson M., Thomas G.N., Batty G.D. (2010). Lymphocyte sub-population cell counts are associated with the metabolic syndrome and its components in the Vietnam Experience Study. Atherosclerosis.

[35] Grunfeld C., Feingold K.R. (1996). Regulation of lipid metabolism by cytokines during host defense. Nutrition.

[36] Barker G., Leeuwenburgh C., Brusko T., Moldawer L., Reddy S.T., Guirgis F.W. (2021). Lipid and lipoprotein dysregulation in sepsis: clinical and mechanistic insights into chronic critical illness. J. Clin. Med..

[37] Tseng Y.H. (2023). Adipose tissue in communication: within and without. Nat. Rev. Endocrinol..

[38] Gao F., Litchfield B., Wu H. (2024). Adipose tissue lymphocytes and obesity. J. Cardiovasc. Aging.

[39] Papayannopoulos V. (2018). Neutrophil extracellular traps in immunity and disease. Nat. Rev. Immunol..

[40] Obama T., Itabe H. (2020). Neutrophils as a novel target of modified low-density lipoproteins and an accelerator of cardiovascular diseases. Int. J. Mol. Sci..

[41] Chaplin D.D. (2010). Overview of the immune response. J. Allergy Clin. Immunol..

[42] Andersen C.J., Murphy K.E., Fernandez M.L. (2016). Impact of obesity and metabolic syndrome on immunity. Adv. Nutr..

[43] Olzmann J.A., Carvalho P. (2019). Dynamics and functions of lipid droplets. Nat. Rev. Mol. Cell Biol..

[44] Nielsen S., Karpe F. (2012). Determinants of VLDL-triglycerides production. Curr. Opin. Lipidol..

[45] O'Sullivan J.A., Bochner B.S. (2018). Eosinophils and eosinophil-associated diseases: an update. J. Allergy Clin. Immunol..

[46] Tabas I., Bornfeldt K.E. (2016). Macrophage phenotype and function in different stages of atherosclerosis. Circ. Res..

[47] Saja M.F., Baudino L., Jackson W.D., Cook H.T., Malik T.H., Fossati-Jimack L. (2015). Triglyceride-Rich lipoproteins modulate the distribution and extravasation of Ly6C/Gr1(low) monocytes. Cell Rep..

